# Supplementary material for: Low PRKAB2 Expression Is Associated with Poor Outcomes in Pediatric Adrenocortical Tumors, and Treatment with Rottlerin Increases the PRKAB2 Level and Inhibits Tumorigenic Aspects in the NCI-H295R Adrenocortical Cancer Cell Line
Source: Cancers (Basel). 2024 Mar 8;16(6):1094. doi: 10.3390/cancers16061094 (PMC10968902; doi:10.3390/cancers16061094)
Supplement: Supplementary file 1 [file cancers-16-01094-s001.zip › cancers-2813896-supplementary.pdf]

# Table S1: Clinical and biological characteristics of patients analyzed in the study.

| Patient | R337H mutation | Age (months) | Gender | Size (cm$^3$) | Weight (g) | Symptoms | Sandrini´s classification | Metastasis | Chemotherapy | Relapse | Event (dead or relapse) | Outcome | Overall survival (months) | Event free survival (months) |
|---|---|---|---|---|---|---|---|---|---|---|---|---|---|---|
| 7 | yes | 22 | female | 6,28 | 10 | Mixed | 1 | No | No | No | No | Alive | 173 | 173 |
| 54 | yes | 101 | female | 527,18 | 580 | Mixed | 2 | No | No | Yes | Yes | Dead | 20 | 20 |
| 58 | yes | 27 | male | 78,81 | 80 | Mixed | 1 | No | No | No | No | Alive | 274 | 274 |
| 105 | yes | 11 | male | 41,19 | 25,9 | Mixed | 1 | No | No | No | No | Alive | 101 | 101 |
| 148 | yes | 20 | male | 36,24 | 60 | Mixed | 2 | No | No | Yes | Yes | Alive | 152 | 7 |
| 150 | no | 137 | female | 1725,9 | 2350 | No symptoms | 4 | yes | Yes | Yes | Yes | Alive | 218 | 53 |
| 164 | yes | 95 | female | 191,29 | 230 | Virilizing | 4 | yes | Yes | Yes | Yes | Dead | 15 | 7 |
| 171 | yes | 92 | female | 284,51 | 380 | Mixed | 1 | yes | Yes | Yes | Yes | Dead | 29 | 9 |
| 195 | no | 13 | male | 28,11 | 40 | Mixed | 1 | No | No | No | No | Alive | 206 | 206 |
| 209 | no | 85 | female | 0,94 | 3,3 | Mixed | 2 | No | No | No | No | Alive | 202 | 202 |
| 235 | yes | 28 | female | 423,63 | 377 | Virilizing | 1 | No | Yes | No | No | Alive | 262 | 262 |
| 361 | yes | 36 | female | 14,06 | 16 | Mixed | 1 | No | No | No | No | Alive | 177 | 177 |
| 383 | yes | 18 | female | 39,23 | 35 | Mixed | 4 | No | No | No | No | Alive | 246 | 246 |
| 387 | yes | 185 | male | 585,76 | 703 | Cushing | 4 | No | No | No | Yes | Dead | 8 | 8 |
| 525 | yes | 33 | male | 1059,08 | 828 | Cushing | 1 | yes | Yes | Yes | Yes | Dead | 7 | 5 |
| 545 | yes | 34 | male | 26,65 | 28,5 | Mixed | 1 | No | No | No | No | Alive | 227 | 227 |
| 686 | yes | 22 | female | 16,57 | 20 | Mixed | 1 | No | No | No | No | Alive | 16 | 16 |
| 698 | no | 145 | female | 82,37 | 53 | Cushing | 4 | No | No | No | No | Alive | 174 | 174 |
| 799 | yes | 66 | female | 376,56 | 351 | Virilizing | 1 | yes | Yes | Yes | Yes | Dead | 15 | 6 |
| 922 | yes | 23 | female | 11,71 | 39 | Mixed | 2 | No | No | No | No | Alive | 174 | 174 |
| 950 | yes | 5 | female | 91,79 | 153 | Mixed | 1 | No | No | No | No | Alive | 176 | 176 |
| 1116 | no | 40 | female | 18,83 | 15 | Mixed | 2 | No | No | No | No | Alive | 163 | 163 |
| 1340 | yes | 82 | male | 59,62 | 108 | Virilizing | 2 | No | No | Yes | Yes | Dead | 7 | 4 |
| 1393 | yes | 21 | female | 56,48 | 84 | Mixed | 1 | No | No | No | No | Alive | 11 | 11 |
| 1455 | yes | 18 | female | 125,52 | 151 | Mixed | 1 | No | No | No | No | Alive | 120 | 120 |
| 1525 | yes | 34 | female | 3,77 | 9 | Virilizing | 1 | No | No | No | No | Alive | 131 | 131 |
| 1993 | yes | 21 | female | 36,61 | 41 | Mixed | 1 | No | No | No | No | Alive | 101 | 101 |
| 2049 | yes | 15 | female | 15 | NA | Virilizing | 1 | No | No | No | No | Alive | 84 | 84 |
| 2329 | no | 157 | female | 16,11 | 154 | No symptoms | 1 | No | No | No | No | Alive | 83 | 83 |
| 2451 | yes | 15 | male | 36,59 | 50 | Mixed | 1 | No | No | No | No | Alive | 58 | 58 |
| 2461 | yes | 18 | female | 17,57 | 19 | Virilizing | 1 | No | No | No | No | Alive | 65 | 65 |
| 2575 | yes | 9 | female | 11,13 | 9 | No symptoms | 2 | No | No | No | No | Alive | 53 | 53 |
| 2637 | yes | 25 | female | 16,24 | 71 | Virilizing | 1 | No | No | No | No | Alive | 54 | 54 |
| 2711 | yes | 35 | female | 520,12 | 402 | Virilizing | 2 | No | No | No | No | Alive | 39 | 39 |
| 5705 | yes | 13 | female | 47,07 | 35 | Mixed | 1 | No | No | No | No | Alive | 133 | 133 |
| 5908 | yes | 12 | male | 109,83 | 102 | Mixed | 1 | No | No | No | No | Alive | 66 | 66 |
| 8202 | yes | 29 | female | 49,57 | 80 | Mixed | 1 | No | No | Yes | Yes | Dead | 16 | 3 |
| 9600 | yes | 17 | female | 25,52 | 20 | Mixed | 1 | No | No | No | No | Alive | 186 | 186 |
| 14606 | yes | 18 | female | 56,01 | 50 | Virilizing | 4 | No | No | No | No | Alive | 36 | 36 |
| 16007 | yes | 16 | female | 15,08 | 16 | Mixed | 1 | No | No | No | No | Alive | 99 | 99 |
| 17903 | no | 21 | male | 196,58 | 75 | No symptoms | 2 | yes | Yes | No | No | Alive | 153 | 153 |
| 23407 | yes | 12 | female | 17,79 | 40 | Mixed | 4 | No | No | No | No | Alive | 113 | 113 |
| 26406 | yes | 92 | female | 73,22 | 102 | Mixed | 1 | No | No | No | No | Alive | 125 | 125 |
| 29091 | yes | 14 | male | 112,21 | 7 | Virilazing | 1 | yes | Yes | Yes | Yes | Alive | 166 | 10 |
| 32804 | yes | 10 | female | 40,27 | 50 | Mixed | 2 | No | No | No | No | Alive | 147 | 147 |
| 35104 | yes | 29 | female | 0,23 | 40 | Mixed | 2 | No | No | No | No | Alive | 50 | 50 |
| 38701 | yes | 16 | female | 192,82 | 150 | Virilazing | 1 | No | No | No | Yes | Dead | 0 | 0 |
| 39499 | yes | 111 | female | 527,18 | 520 | Virilazing | 2 | No | Yes | Yes | Yes | Dead | 9 | 3 |
| 40007 | yes | 15 | female | 12 | 70 | Mixed | 2 | No | No | No | No | Alive | 109 | 109 |
| 41203 | yes | 187 | female | 596,22 | 634 | Mixed | 1 | No | No | No | No | Alive | 133 | 133 |
| 41503 | no | 17 | female | 344,2 | 395 | Virilazing | 3 | No | No | No | No | Alive | 140 | 140 |
| 44608 | yes | 22 | female | 52,3 | 84 | Virilazing | 3 | No | No | Yes | Yes | Alive | 28 | 23 |
| 47009 | yes | 16 | female | 58,25 | 68 | Mixed | 1 | No | Yes | No | No | Alive | 80 | 80 |

| | | | | | | | | | | | | | | |
|---|---|---|---|---|---|---|---|---|---|---|---|---|---|---|
| 48705 | yes | 19 | female | 456,67 | 228 | Virilazing | 1 | No | Yes | No | No | Alive | 130 | 130 |
| 48708 | yes | 13 | female | 28,87 | 25 | Mixed | 2 | No | Yes | No | No | Alive | 94 | 94 |
| 53201 | yes | 17 | female | 13,71 | 15 | Mixed | 4 | No | Yes | Yes | Yes | Alive | 179 | 3 |
| 59603 | yes | 12 | female | 13,69 | 15 | Mixed | 1 | No | No | No | No | Alive | 120 | 120 |
| 61301 | no | 13 | female | 313,8 | 340 | Mixed | 1 | No | No | No | No | Alive | 172 | 172 |
| 63205 | yes | 19 | female | 313,8 | 340 | Virilazing | 1 | yes | Yes | No | Yes | Dead | 7 | 7 |
| 68705 | yes | 25 | female | 39,43 | 65 | Mixed | 3 | No | No | No | No | Alive | 123 | 123 |
| 70203 | yes | 10 | female | 15,32 | 35 | Mixed | 1 | No | No | No | No | Alive | 153 | 153 |
| 74099 | yes | 44 | female | 40,27 | 20 | Mixed | 1 | No | No | No | No | Alive | 203 | 203 |
| 74804 | yes | 52 | male | 621,75 | 810 | Mixed | 1 | No | Yes | No | No | Alive | 143 | 143 |
| 76202 | yes | 5 | male | 17,42 | 20 | Virilazing | 1 | No | No | No | No | Alive | 148 | 148 |
| 81108 | yes | 38 | female | 0,45 | 6 | Mixed | 2 | No | No | No | No | Alive | 90 | 90 |
| 84001 | yes | 64 | female | 34,29 | 55 | Mixed | 1 | No | No | No | No | Alive | 174 | 174 |

**Table S2: Primers sequences used to evaluate gene expression of the *PRKAB2, TSC1, STK11*, and *GUS* genes.**

| Gene | Primer sequence |
|---|---|
| *PRKAB2* | Forward: 5′AAATCCCCACCCATCCTTC3 |
| | Reverse: 3′GCATAACATGGTTGGGCTCA5 |
| *TSC1* | Foward:5′AGCCAATGATGGAGCATGTG3′ |
| | Reverse: 3′ GGCACACTCGATCACAACAT5′ |
| *STK11* | Foward: 5′CTTCTTTGGCGACGAGTCAA3′ |
| | Reverse: 3′ATGATGGGATCTGTCGAGG5 |
| *GUS* | Foward: 5′GAAAATATGTGGTTGGAGAGCTCATT3′ |
| | Reverse: 3′CCGAGTAAGATCCCCTTTTTA5′ |

**Methods (Supplemental material)**

**Cell line**

The NCI-H295R [H295R] adrenocortical carcinoma cell line (ACTC® CRL-2128™), was cultured in DMEM-F12 medium supplemented with 2.5% NuSerum (CORNING, Teo Oak Park, Bedford, MA, USA), 1% ITS (GIBCO, Grand Island, NY 14072, USA), 60 mg/mL penicillin, and 100 µg/mL streptomycin (GIBICO, Grand Island, NY 14072, USA) in humid atmosphere containing 5% $CO_2$, at 37 °C. An STR test was performed to confirm lineage authenticity. The H295R cell line present an inactivation mutation in the ***TP53*** gene and an activating mutation in the ***CTNNB1*** gene (c.T133C:p.S45P). These cells are the only commercially available cell line that was confirmed as a human ACT.

**Treatment with Rottlerin**

NCI-H295R cells were treated with Rottlerin (protein kinase C delta inhibitor: 1610, Tocris Bioscience, Atlantic Road, Bristol) for 6-72h according to each specific assay. A 1 mmol/L Rottlerin stock solution was prepared in dimethylsulfoxide (DMSO), and aliquots of this solution were stored at -20 °C. The $IC_{50}$ and $IC_{25}$ values were established by using the Compusyn software (ComboSyn Inc.)

**RNA extraction and gene expression by real-time PCR (RT-qPCR)**

ACT,non-neoplastic adrenal and NCI-H295R samples were subjected to total RNA extraction by using the TRIZOL® reagent (InvitrogenInc, Carlsdab, CA, USA). cDNA was synthesized with the High Capacity® kit (AppliedBiosystems, Foster City, CA, USA) according to the manufacturer. For gene expression analysis, NCI-H295R cells were treated with Rottlerin for 48h, followed by RNA extraction and cDNA synthesis by using the same protocol mentioned above. Gene expression was analyzed by the qRT-PCR technique. The *GUSB* gene was used as an endogenous control, and the mean gene expression values of six non-neoplastic adrenal samples were used as calibrator. The employed primers are shown in Supplementary Table 1. Experiments were carried out on the QuantStudio™ 12K Flex equipment (Applied Biosystems, Foster City, CA, USA), by using the SYBR Green® and GoTaq Probe® PCR Master Mix (Applied Biosystems, Foster City, CA, USA): *GUSB* (4326320E), *STAR* (00986559), *MYCN* (00232074), *TCF7L2* (Hs01009044), *LEF1* (Hs01547250), *AXIN2*

(00610344), *GSK3B* (Hs01047719), and *AKT1* (00178289). Relative gene expression was quantified with the $2^{-\Delta\Delta CT}$ equation (Livak & Schmittgen, 2001). The standard curve for each primers was constructed to determine efficiency, by considering an efficiency of 90–100% (slopes between -3.6 and -3.3). All the samples were analyzed in at least three independent experiments performed in triplicate.

**Cell viability assay**

Cell viability was analyzed by using the CellTiter Glo® Cell Luminescent Cell Viability kit (Promega, Madison, WI, USA) according to the manufacturer's protocol. Briefly, NCI-H295R cells were cultivated in a 96-well plate at a density of $2 \times 10^4$ cells/well; and treated with 4.5 µM ($IC_{50}$) or 2.25 µM ($IC_{25}$) of Rottlerin for 24, 48, or 72 h. After treatment, the luminescence was measured using a SpectraMax® L. At least three independent experiments were performed for each concentration and time in triplicate.

**Clonogenic Assay**

NCI-H295R cells were treated with Rottlerin at 4.5 µM ($IC_{50}$) or 2.25 µM ($IC_{25}$) in 25-$cm^3$ bottles, at a confluence of 4 x $10^6$, for 24 or 48 h. Then, cells previously treated with Rottlerin (2.5 x $10^2$ cells/well) were seeded in a 12-well plate and cultured with normal medium for additional four weeks for colony formation. Medium was changed every 48 h. Colonies were then fixed with methanol,stained with Giemsa and counted. Only colonies with more than 50 cells were considered for analysis. The assays were analyzed in at least three independent experiments performed in triplicate.

**Transwell Assay**

NCI-H295R cells were treated with 4.5 µM ($IC_{50}$) or 2.25 µM ($IC_{25}$) of Rottlerin in 25-$cm^3$ bottles, at a confluence of 4 x $10^6$, for 48 h. Then, 2 x $10^5$ cells suspended in DMEM-F12 medium without supplement were seeded per insert. Supplemented medium was added to the 24-well plate, below the insert, to stimulate cell migration. Cells were allowed to migrate for 72 h and then fixed with methanol and stained with Giemsa 1% (diluted in phosphate buffer). The migrated cells were photographed under an inverted microscope in three randomly selected fields along the scraped line and measured by using the ImageJ software version 1.48 (NIH, Washington, USA). The assays were repeated in three independent experiments, in triplicate.

**Hormone measurement**

For hormone quantification, 5 x $10^5$ NCI-H295R cells/well ware seeded in a six-well plate and after24 h were treated with DMSO or 4.5 µM ($IC_{50}$) or 2.25 µM ($IC_{25}$) of Rottlerin for 48 h. After that, the culture medium was collected and immediately stored at -80 °C. Cortisol, D4-androstenedione (D4), and testosterone concentrations were determined by radioimmunoassay (RIA) as previously described (DOI:10.1210/jcem.74.1.1309366). All the hormones were measured in triplicate and the mean was normalized to the cell viability effects in the same treatment conditions.

**Western blotting**

For western blotting analysis, NCI-H295R cells were treated with 4.5 µM ($IC_{50}$) or 2.25 µM ($IC_{25}$) of Rottlerin for 6, 12, 24, or 48 h, followed by protein extraction. between 20-40 µg of proteins was separated by SDS-PAGE electrophoresis and membrane incubated with 1:1000 anti-AMPKβ2 primary antibodies (4148S), 1:1000 p-AMPK (#2535), 1:1000 AMPKα-total (#2532), 1:1000 p-mTOR (#2971), and 1:1000 mTOR-total (#2983), obtained from Cell Signaling Technology, Danvers, MA, USA, and 1:2500 anti-GAPDH (sc-47724) and 1:500 MAP LC3B (sc-271625), obtained from Santa Cruz Biotechnology (Santa Cruz, California, USA). Anti-mouse IgG 1:5000 (#7076) and anti-rabbit IgG 1:5000 (#7074) secondary antibodies were obtained from Cell Signaling Technology, Danvers, MA, USA. A molecular weight marker (MW) and the protein of interest run on the same gel. The detection of the MW is done by colorimetry, just to control the size of the band of interest. The protein was detected by chemiluminescence using the ECL™ Western blotting Analysis System (Amersham GE Healthcare, Buckinghamshire, UK). Intensity was quantified by using the ImageJ software version 1.48 (NIH, Washington, USA), relativized by the control, and normalized by an endogenous protein (GAPDH). Membrane stripping was performed a maximum of three times using Restore Western Blot Stripping buffer (Thermo Scientific, Illinois, USA).

**RNA sequencing (RNAseq)**

To investigate the role played by Rottlerin in NCI-H295R cells, global gene expression was evaluated by RNAseq analysis in cells treated with Rottlerin ($IC_{50}$) or control (DMSO) for 48 hours. The Illumina Stranded mRNA Sample Preparation kit (Illumina Inc) was used to prepare the samples. The generated libraries were evaluated by using the Tapestation equipment (Agilent Technologies) and quantified on a Qubit fluorimeter with the Qubit dsDNA BR Assay kit (Life

Technologies). Clustering and sequencing were performed on the Illumina NovaSeq 6000 equipment by using the NovaSeq SP Kit (200 cycles) (Illumina Inc), to generate 800 million reads per sequencing. The data quality of the two samples (Rottlerin at $IC_{50}$ and DMSO) was evaluated by using the FastQC (0.11.3) and MultiQC (v1.10) programs, which provide information about basic sequence statistics, quality, GC content, sequence size distribution, sequence duplication levels, and over-represented sequences. For alignment, mapping, and counting of the sequences against the reference genome, the STAR aligner (v2.7.8a) was used. From the count of reads in each sample, the R programming language (v4.1.2) was applied; more specifically, the DESeq2 package was employed, which enabled differential expression between the different conditions of the experiment to be analyzed. On the basis of Log Fold-Change (LogFC), the differentially expressed genes (DEGs) were separated into high and low expression and subsequently enriched for deregulated pathways through the enrichment platform (https://maayanlab.cloud/Enrichr/). Sequencing was carried out at the Institute for Cancer Research (IPEC), Guarapuava – PR.

**Statistical analysis**

All data obtained from functional assays and patients' samples evaluations were analyzed by using the GraphPadPrism 8.0 (GraphPad Software, San Diego, CA, USA) or SPSS 20.0 (SPSS Inc. Chicago, USA) statistical programs. At least three independent experiments were carried out in triplicate and the average of the experiments was considered. The Mann-Whitney test was used to analyze patients' gene expression and clinical characteristics. The One-way and Two-way – ANOVA tests were used for functional tests, with a post-hoc Bonferroni test were used for functional; $p < 0.05$ was adopted as the level of statistical significance. Analysis of five-year event-free survival (with relapse and/or death from any cause being considered an unfavorable event) and five-year overall survival (with death being considered an unfavorable event) rates was based on Kaplan-Meier curves and log-rank test. The median *PRKAB2* expression was used as the cut-off point for high or low expression. The multivariate test using the Cox regression model was applied to assess independence of the factors in the predictive capacity.

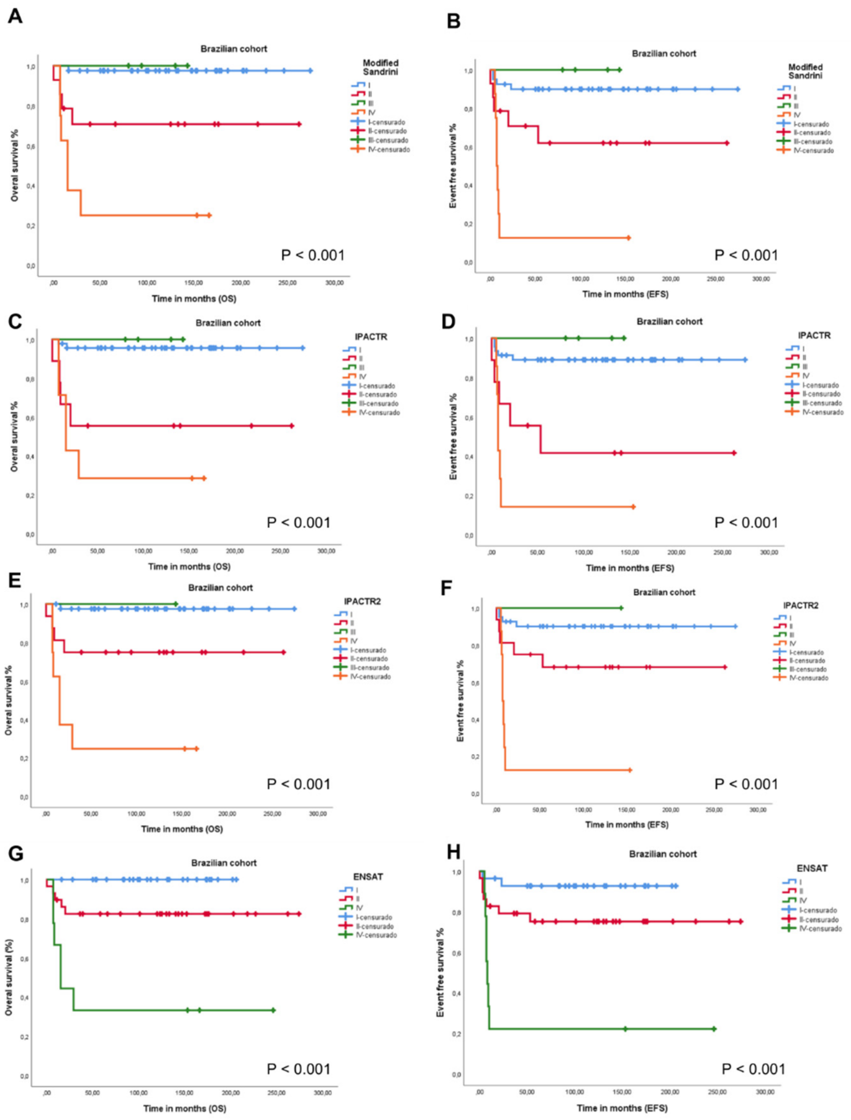


**Supplementary Figure S1.** Kaplan-Meier curves and log-rank test show a lower survival in stage IV patients in our cohort. (A) OS ENSAT cohort (p <0.001) Stage I (n=28) II (n= 29), IV (n =9). (B) EFS ENSAT (p<0.001) stage I (n=28), II (n=29) and IV (n=9). (C) OS IPACTR stage I (n=46), II (n=9), III (n=4), and IV(n=7). (D) EFS IPACTR stage I (n= 46), II (n= 9), III (n= 4), and IV (n= 7). (E) OS IPACTR2 (p < 0.001), Stage I (n=41), II (n=26), III (n=1), and IV (n=8). (F) EFS IPACTR2 (p<0.001), stage I (n=41), II (n=16), III (n=1), and IV (n=8). (G) Modified Sandrini (p < 0.001) Stage I (n=40), II (n=14), III (n=4) and IV (n=8). (H) Modified EFS Sandrini (p< 0.001) stage I (n=40), II (n=14), III (n=4), and IV (n=8).

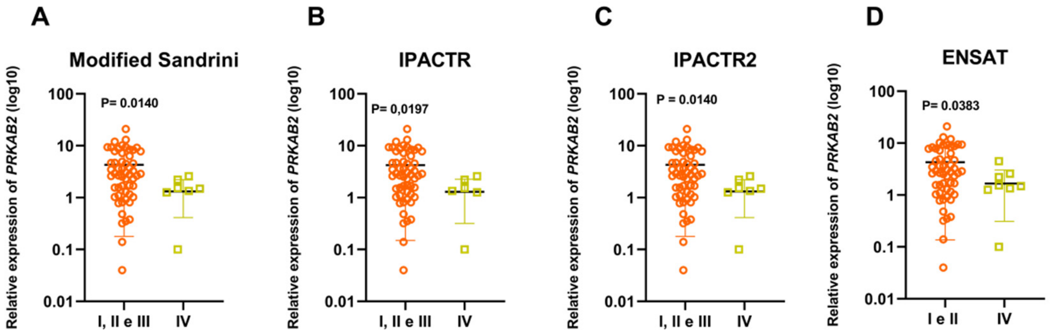


**Supplementary Figure** S2. (A) ENSAT patients in stages I, II, and III have lower PRKAB2 gene expression (p= 0.03); (B) IPACTR patients in stages I, II, and III showed lower expression of the PRKAB2 gene when compared to patients in stage IV (p=0.019); (C) IPACTR2 Patients with stage I, II and III showed lower expression of the PRKAB2 gene when compared to controls (p = 0.014); (D) Modified Sandrini, patients with stages I, II and III lower PRKAB2 gene expression when compared to stage IV (p= 0.0197) (Mann-Whitney test).
