# Supplementary figures and images for: Low PRKAB2 Expression Is Associated with Poor Outcomes in Pediatric Adrenocortical Tumors, and Treatment with Rottlerin Increases the PRKAB2 Level and Inhibits Tumorigenic Aspects in the NCI-H295R Adrenocortical Cancer Cell Line
Source: Cancers (Basel). 2024 Mar 8;16(6):1094. doi: 10.3390/cancers16061094 (PMC10968902; doi:10.3390/cancers16061094)

# PRKAB2

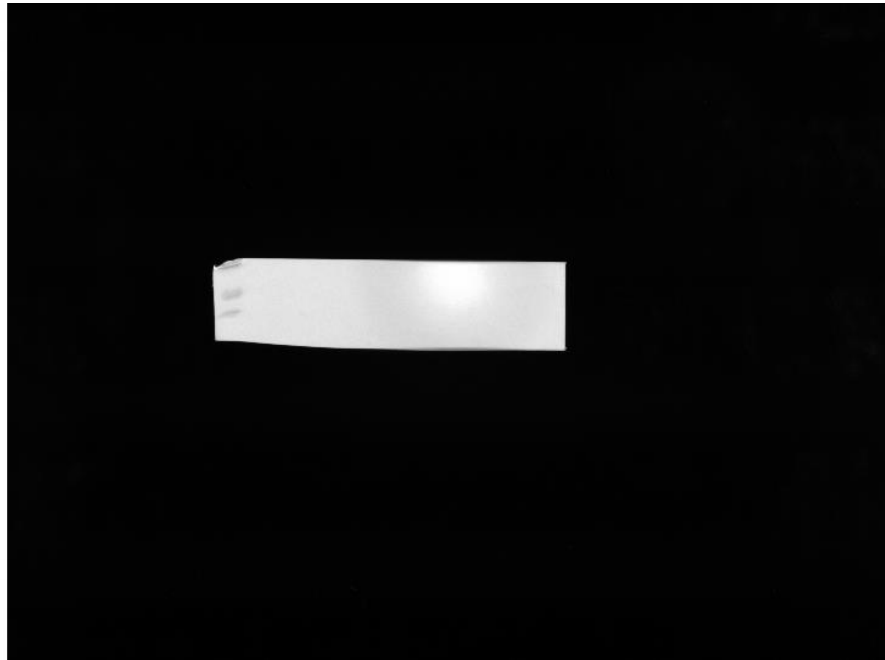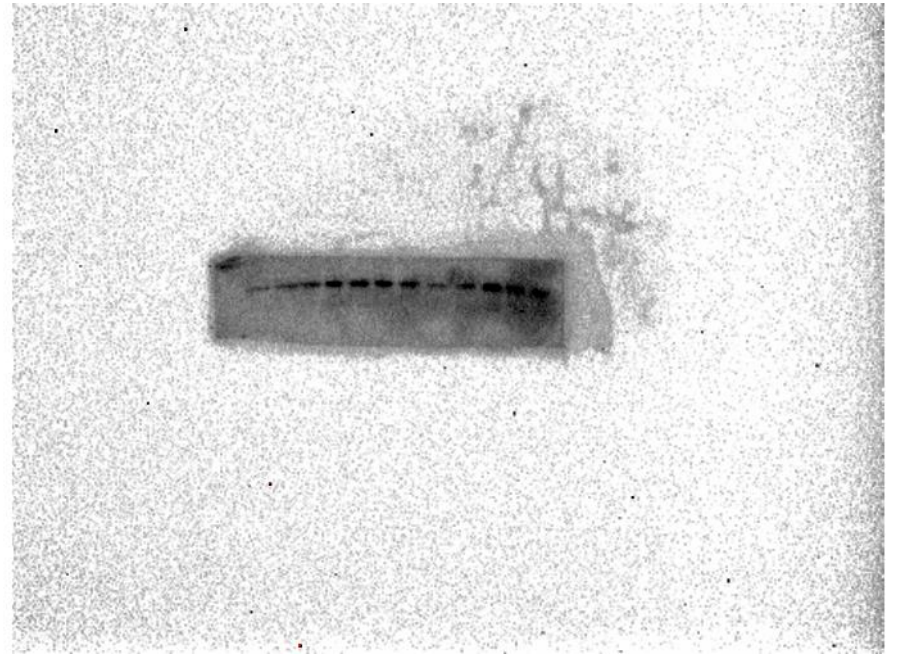

AMPK total

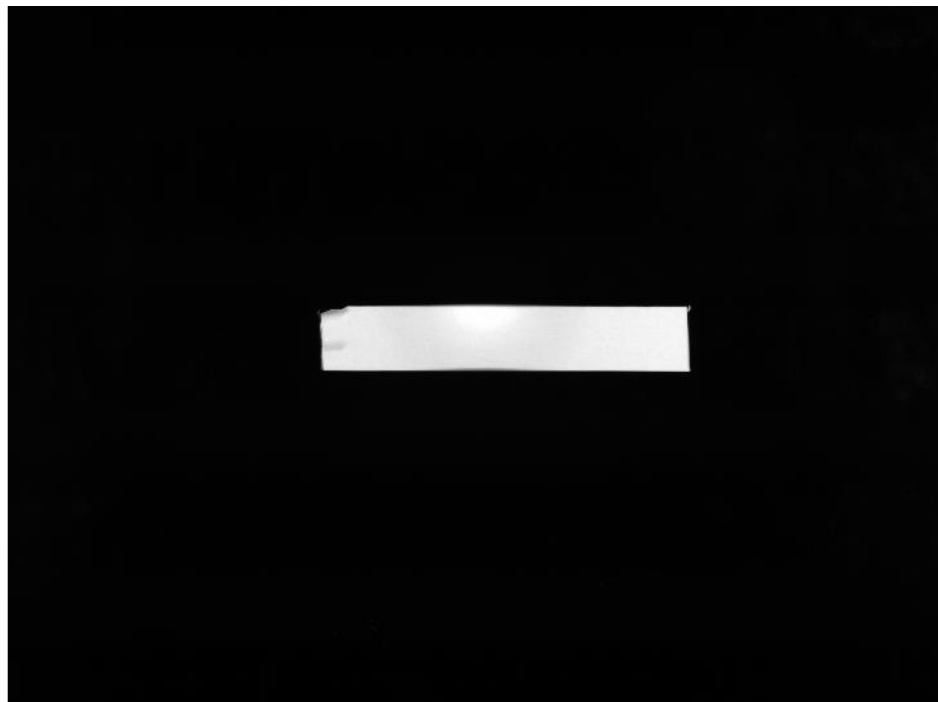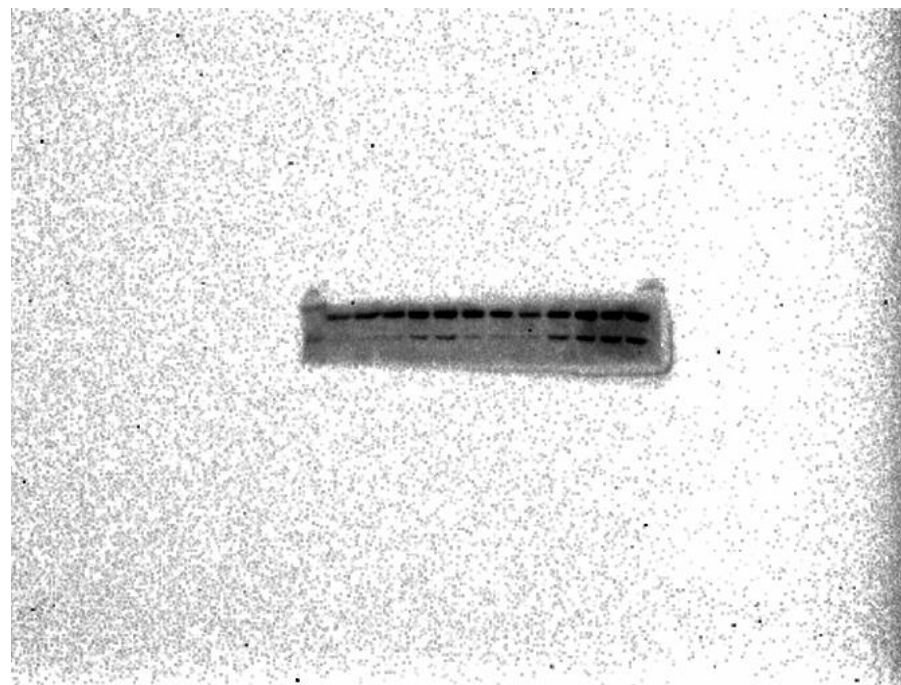

# P-AMPK

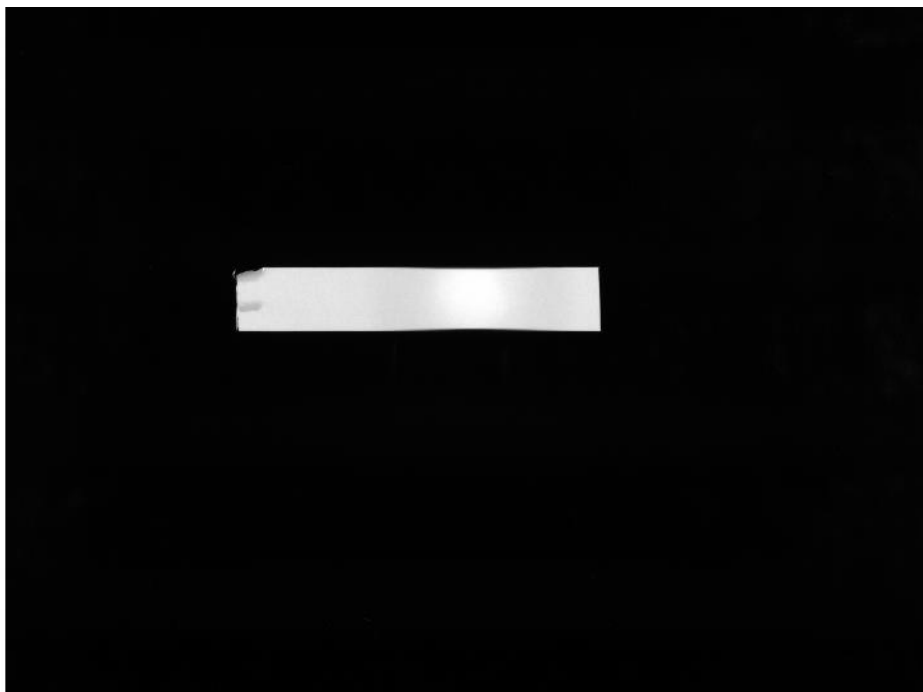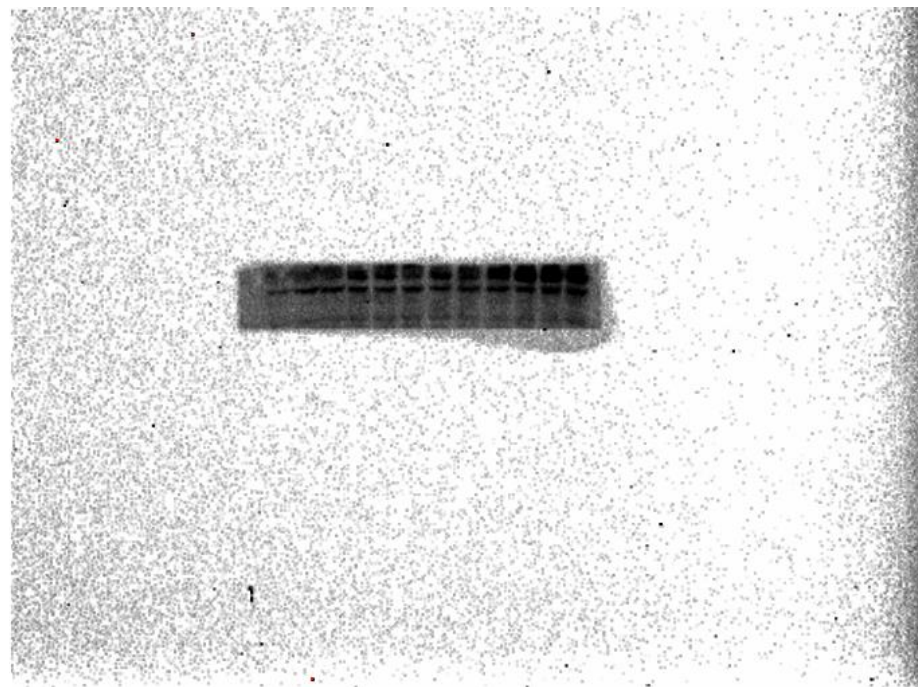

# GAPDH

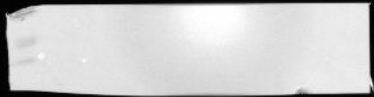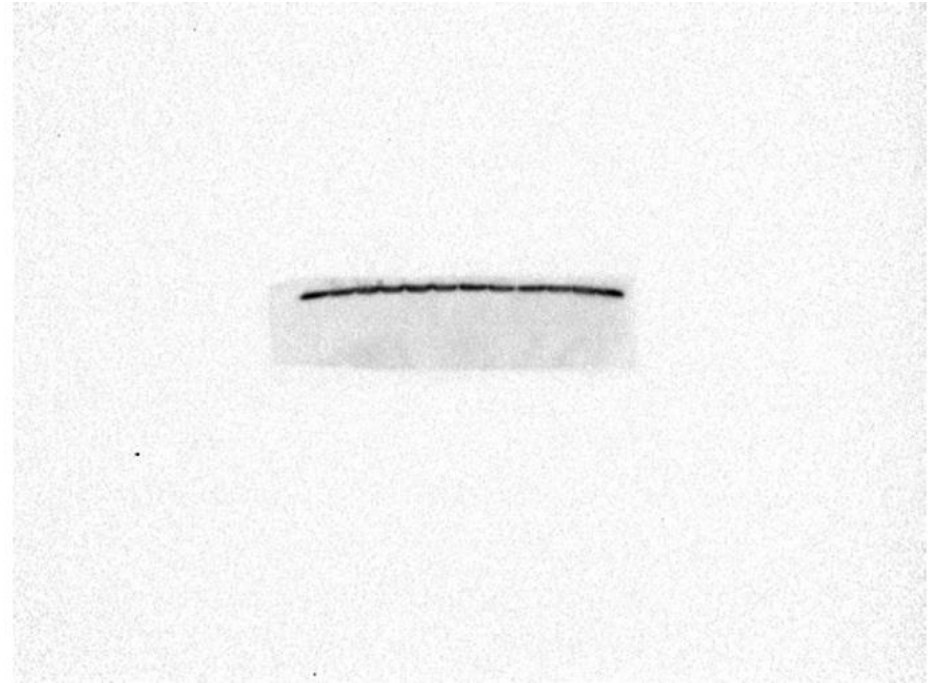

# ACC

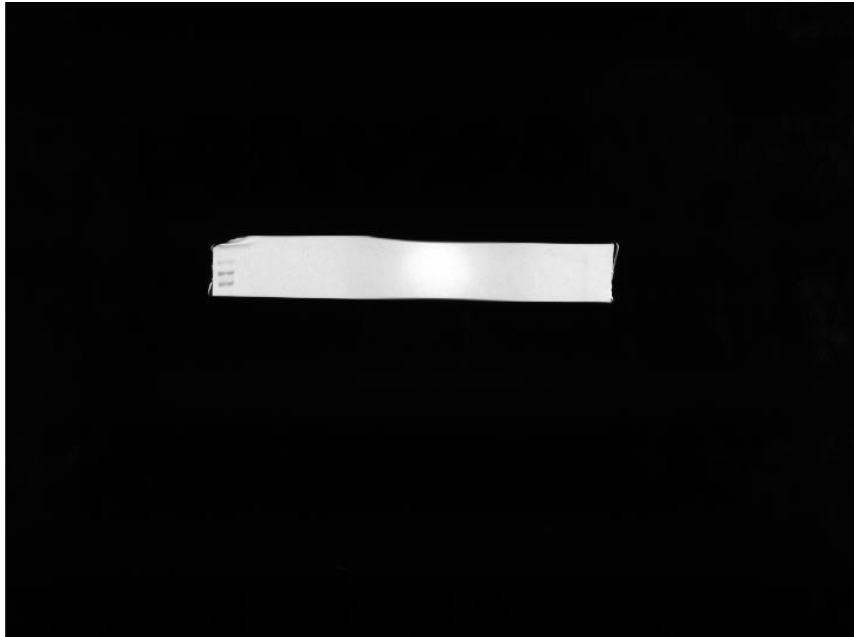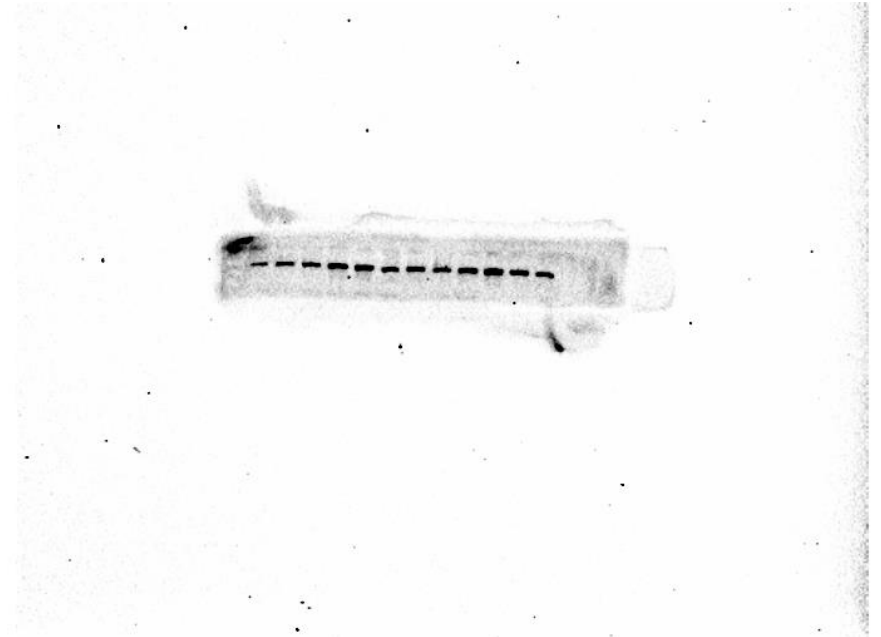

# MTOR-TOTAL

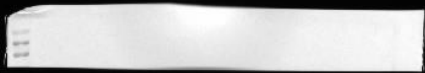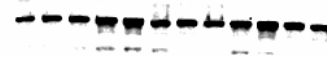

# P-MTOR

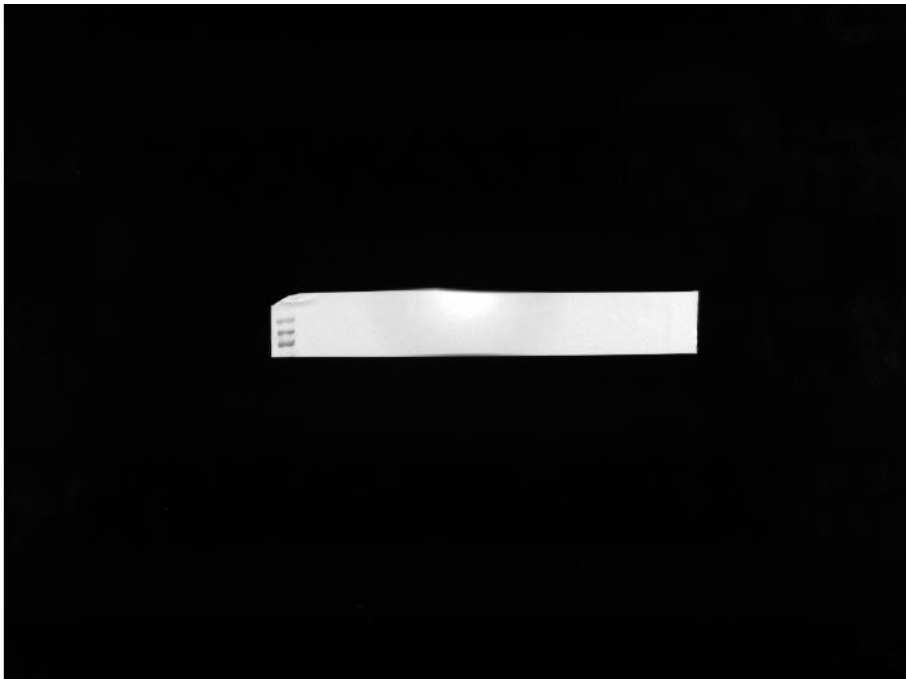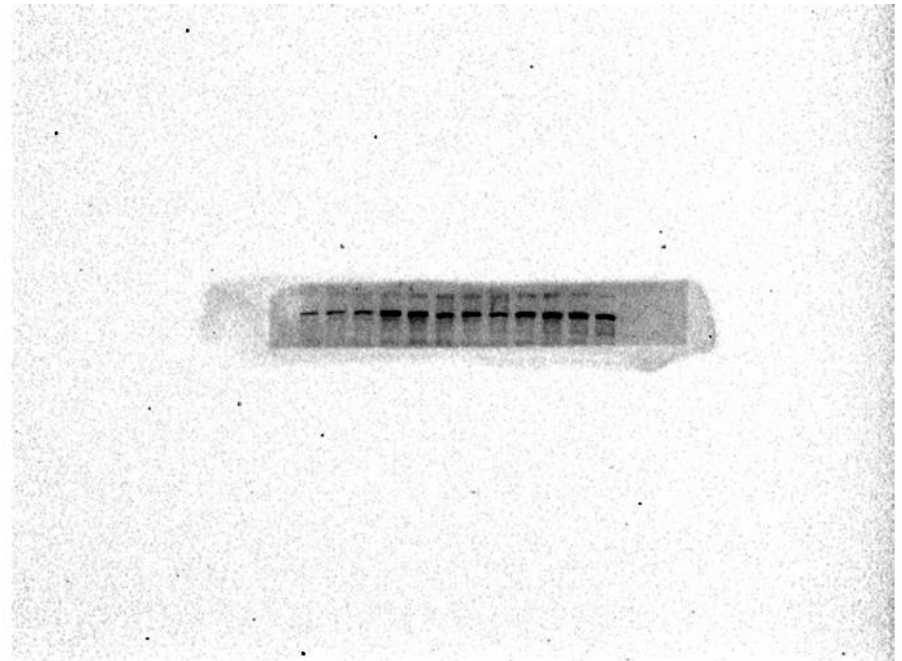

# GSK3B

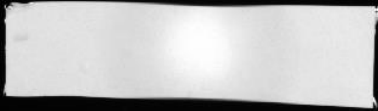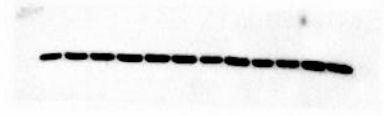

# P-GSK3B

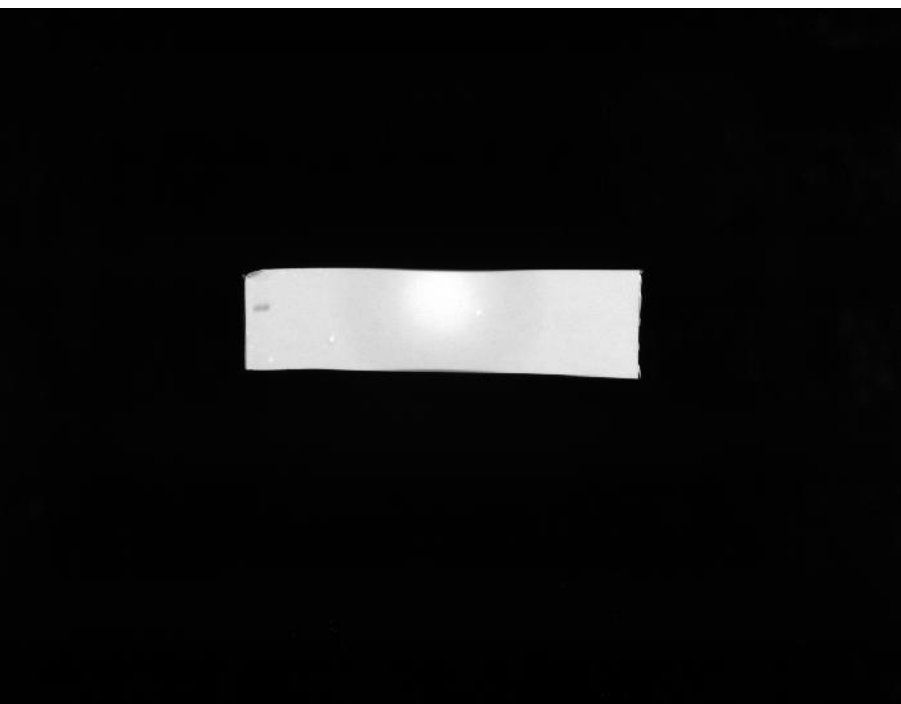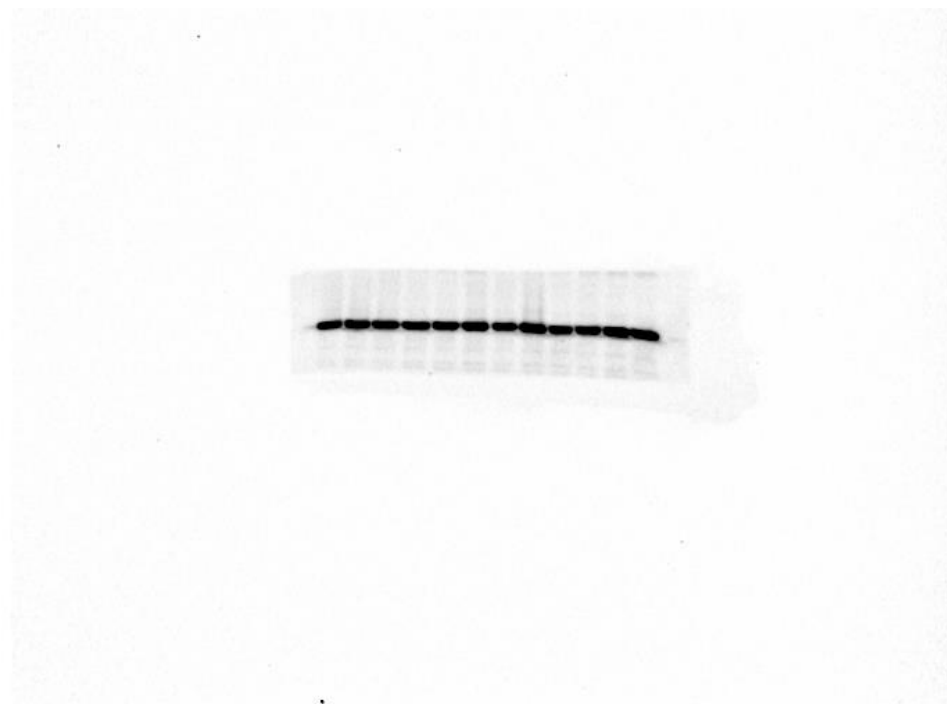

# SKP2

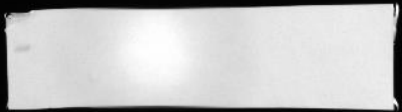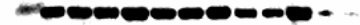

# GAPDH

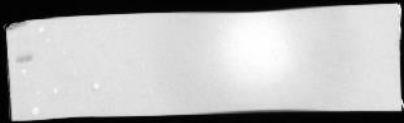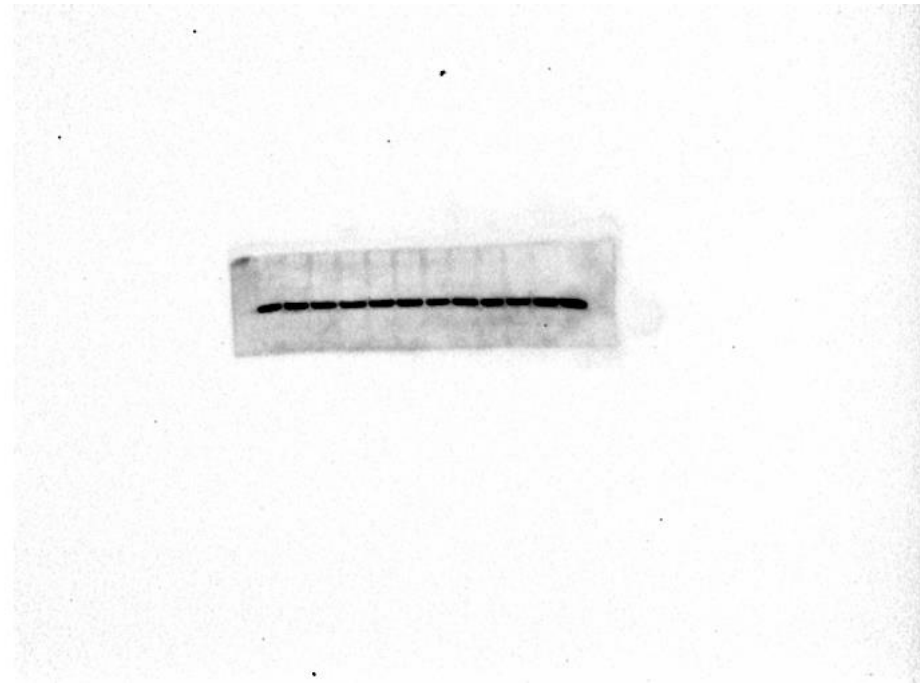

# LC3B

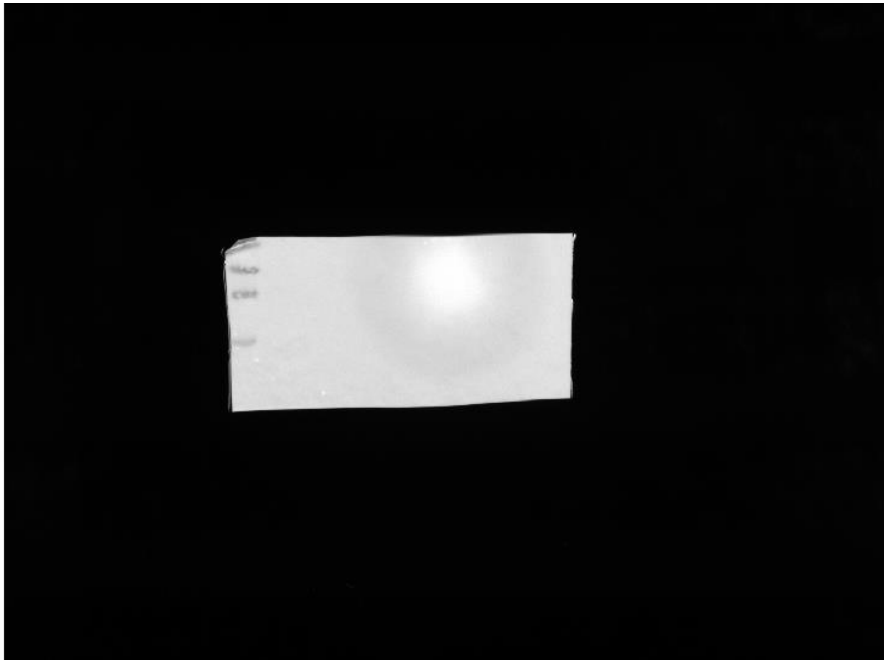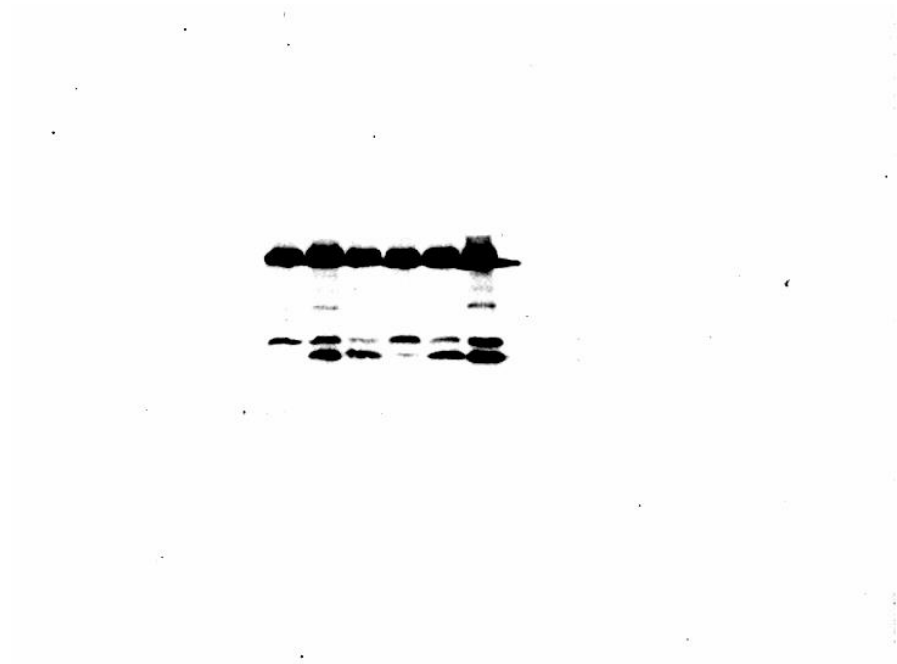

# BAX

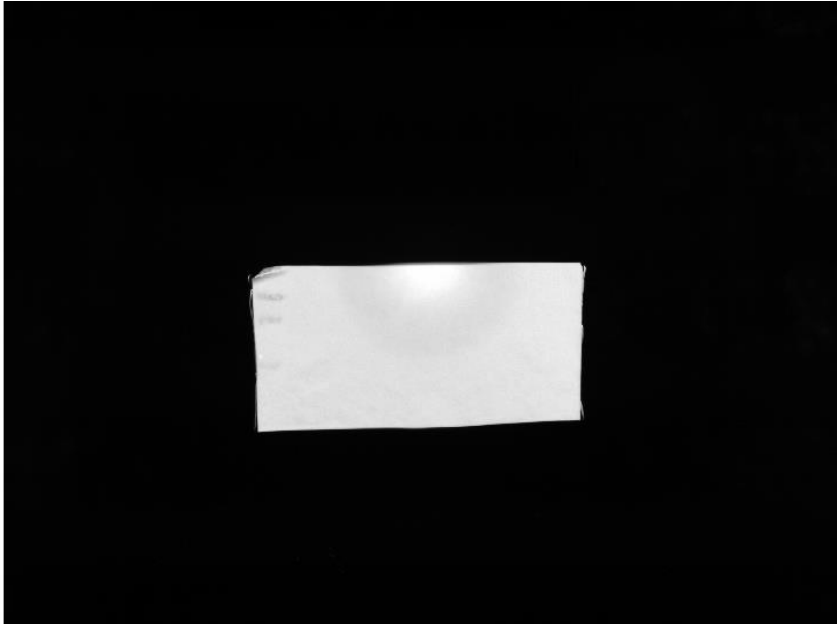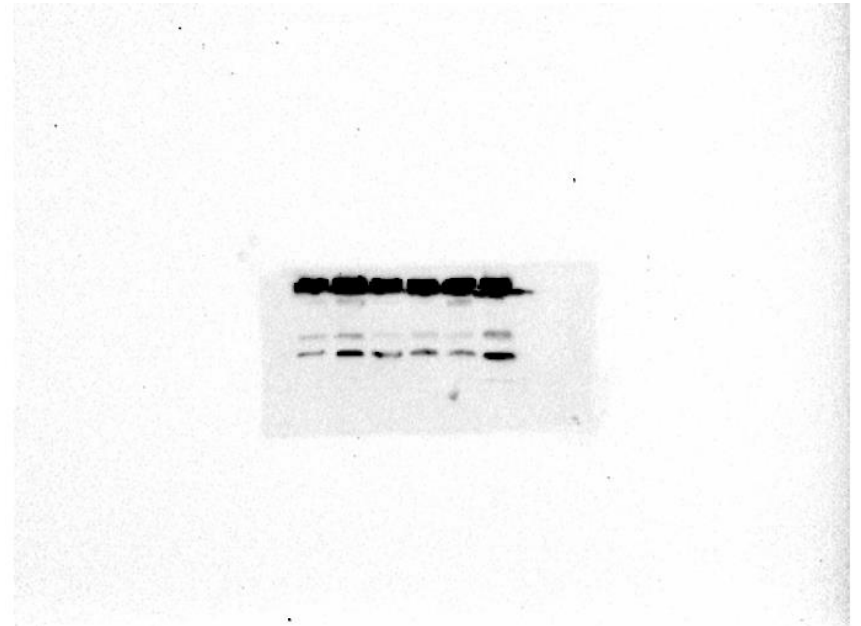

# GAPDH

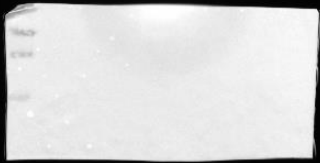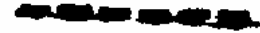

Supplement: Supplementary file 1 [file cancers-16-01094-s001.zip › cancers-2813896-File S1.pdf]
